# Pituitary tumours without distinct lineage differentiation express stem cell marker SOX2

**DOI:** 10.1007/s11102-024-01385-0

**Published:** 2024-03-14

**Authors:** Nèle F. Lenders, Tanya J. Thompson, Jeanie Chui, Julia Low, Warrick J. Inder, Peter E. Earls, Ann I. McCormack

**Affiliations:** 1 Department of Endocrinology, St Vincent’s Hospital, Sydney, NSW Australia; 2 Garvan Institute of Medical Research, Level 4, 384 Victoria St, Darlinghurst, NSW Australia; 3 St Vincent’s Clinical School, University of New South Wales, Sydney, NSW Australia; 4 Department of Anatomical Pathology and Cytopathology, St Vincent’s Pathology, Sydney, NSW Australia; 5 Department of Diabetes and Endocrinology, Princess Alexandra Hospital, Brisbane, QLD Australia; 6 Faculty of Medicine, the University of Queensland, Brisbane, QLD Australia

**Keywords:** Pituitary, Tumour, Immunohistochemistry, RT-PCR

## Abstract

**Context:**

The recent WHO 2022 Classification of pituitary tumours identified a novel group of ‘plurihormonal tumours without distinct lineage differentiation (WDLD)’. By definition, these express multiple combinations of lineage commitment transcription factors, in a monomorphous population of cells.

**Objectives:**

To determine the expression of stem cell markers (SOX2, Nestin, CD133) within tumours WDLD, immature PIT-1 lineage and acidophil stem cell tumours, compared with committed cell lineage tumours.

**Methods:**

Retrospective evaluation of surgically resected pituitary tumours from St Vincent’s Hospital, Sydney. Patients were selected to cover a range of tumour types, based on transcription factor and hormone immunohistochemistry. Clinical data was collected from patient files. Radiology reports were reviewed for size and invasion. Samples were analysed by immunohistochemistry and RT-qPCR for SF-1, PIT-1, T-PIT, SOX2, Nestin and CD133. Stem cell markers were compared between tumours WDLD and those with classically “mature” types.

**Results:**

On immunohistochemistry, SOX2 was positive in a higher proportion of tumours WDLD compared with those meeting WHO lineage criteria, 7/10 v 10/42 (70 v 23.4%, *p* = 0.005). CD133 was positive in 2/10 tumours WDLD but 0/41 meeting lineage criteria, *P* = 0.003. On RT-qPCR, there was no significant difference in relative expression of stem cell markers (SOX2, CD133, Nestin) between tumours with and WDLD.

**Conclusions:**

Our study is the first to biologically characterise pituitary tumours WDLD. We demonstrate that these tumours exhibit a higher expression of the stem cell marker SOX2 compared with other lineage-differentiated tumours, suggesting possible involvement of stem cells in their development.

## Introduction

During pituitary development, undifferentiated stem cells of the Rathke’s pouch generate all committed cell lineages of the anterior pituitary gland, following downregulation of stem cell transcription factors and upregulation of lineage specific transcription factors [1–3]. T-box transcription factor 19 (TBX19, T-PIT) expression occurs with development of corticotroph cells, steroidogenic factor 1 (NR5A1, SF-1) with development of gonadotroph cells and pituitary specific positive transcription factor 1 (POU1f1, PIT-1) with development to PIT-1 cell lineage, encompassing somatotroph, somatomammotroph, lactotroph and thyrotroph cells [1–3]. Committed progenitor cells are proliferative and give rise to hormone producing cells; which expand rapidly after birth. Ability of pituitary stem cells to proliferate and differentiate decreases with age into adulthood [1].

The role of stem cells in pituitary tumorigenesis has been the subject of debate. Pituitary tumours have classically been thought to be monoclonal in origin, based on evidence from X-chromosome inactivation studies and allelotype analyses [4, 5]. However, there is growing recognition of tumour heterogeneity and cell plasticity, observed in certain tumours, between primary and metastatic disease, and in hormonal transformation of tumour types [6, 7]. Advances in single cell technologies have facilitated a rapidly expanding body of evidence in this area, through spatially resolved cellular analyses and imaging [8].

Stem cells have been demonstrated to play a significant role in a number of malignancies such as leukaemia and solid organ cancers like breast cancer [9]. . The cancer stem cell theory proposes that neoplasia arise from tumour stem cells, maintain themselves by self-renewal and can “differentiate” to form different elements of the tumour mass [9]. The existence of cancer stem cells would account for observed intra-tumour heterogeneity; persistence and recurrence following apparent gross total resection and poor response to cytotoxic agents and radiotherapy, due to slow cell cycling and high expression of DNA repair proteins [9]. Several human pituitary tumour studies have isolated cells fulfilling some or all of the tumour stem cell criteria, specifically: ability to grow cell spheres in vitro; expression of stem cell markers and pituitary specific markers; ability to self-renew and to differentiate to form lineage specific hormone producing cells; cytotoxic resistance and in vitro tumourigenicity [9–15]. Within these studies, various pituitary stem cell markers have been studied. Three of the most consistently examined stem cell markers were SOX2, CD133 and Nestin [9–15]. SOX2 acts as a pluripotency inducing factor, which has an important role in pituitary stem cell multipotency [16]. Recent single nucleus transcriptomic data has demonstrated expression of SOX2 and SOX9 in uncommitted pituitary stem cells, whereas Nestin was expressed in a proportion of committed cells and was not universally expressed in pituitary stem cells [17].

The 2017 WHO Classification heralded a paradigm shift in the histopathological diagnosis of pituitary tumours, now based on cellular lineage, determined by transcription factor and hormonal immunohistochemistry [18]. Clinical application of transcription factor immunohistochemistry soon highlighted shortcomings within the new classification [19]. Notably, a previously undescribed group of tumours was identified, characterised by co-expression of two or more lineage-specific transcription factors. Co-expression of SF-1 and PIT-1 has also been reported in two case reports, wherein authors suggested that such tumours may represent poorly differentiated stem cell tumours [20, 21]. We have recently published on transcription factor analysis of 172 patients with pituitary tumours, in which we found 14 (8%) expressed both SF-1 and Pit-1 [19].

In the 2022 WHO Classification, a new category of pituitary tumours was named “Tumours without distinct lineage differentiation (WDLD)”, incorporating the newly defined “Plurihormonal tumours” along with previously defined “Null cell tumours”. The newly defined “Plurihormonal tumour” type was described as “very rare”, comprising a monomorphous population of cells that display features of multiple lineages [22]. The biology and prevalence of these tumours remains to be determined [20, 21, 23]. Although not classified as WDLD, another important change in the new classification was the distinction between mature plurihormonal PIT-1 lineage and immature PIT-1 lineage tumours [22].

Herein, we examined the expression of stem cell markers within WDLD, immature PIT-1 lineage and acidophil stem cell tumours, compared to classically ‘mature’ tumours with committed cell lineage. Additionally, we investigated the relationship between these tumours and markers of proliferation and invasion.

## Materials and methods

### Study design

This was a retrospective evaluation of surgically resected tumours from St Vincent’s Hospital, Sydney (2018_ETH00188). Ethical approval was obtained, complying with the Declaration of Helsinki. Patients were not required to provide consent, due to the retrospective and de-identified nature of our study.

### Patient selection and data collection

Patients were selected (non-consecutively) to cover a range of different histopathological tumour types based on transcription factor immunohistochemistry (IHC): tumours expressing both SF-1 and PIT-1; tumours expressing SF-1 only; tumours expressing PIT-1 only; tumours expressing T-PIT only; PIT-1 lineage tumours (immature and mature plurihormonal) and normal pituitary tissue (control). Tumours expressing both SF-1 and PIT-1 were only selected if there was strong co-expression of each transcription factor. Patients were selected based on availability of tissue for immunohistochemistry and RT-qPCR analysis, favouring more recently operated tumours for sample quality. Clinical data was collected including patient demographics, presentation, tumour type and interventions (numbers of surgeries, radiotherapy, medical therapies). Radiology reports were reviewed for tumour size, cavernous sinus (CS) or sphenoid sinus (SS) invasion and remnant disease post-operatively. Histopathology reports were reviewed for results of haematoxylin and eosin (H&E) staining, hormonal immunohistochemistry (IHC), Ki67 and mitotic count.

### Immunohistochemistry

Whole tumour Formalin-Fixed Paraffin-Embedded (FFPE) sections were cut and stained with antibodies for SF-1, PIT-1, T-PIT, SOX2, Nestin and CD-133. Antibody selection is summarised in Table 1, and was based on previous experience in pituitary tissue where available. Images were digitised with Roche DP200 slide scanner. Sections were reported for percentage of positive cells, staining intensity (strong, moderate, weak) and distribution with the assistance of tissue immunohistochemistry analysis software (Indica Labs, Halo) and three pituitary pathologists (JC, JL and PE). Stem cell markers were considered to be positive where ≥1% cells were positive.

**Table 1:** Antibodies for immunohistochemistry and primers for RT-qPCR analysis

Gene	Protein	IHC antibody	IHC control	RT-qPCR Assay	References
SOX2, 3q26.33	SOX2	Cell Signalling, Danvers: Sox2 (D1C7J) XP^®^ Rabbit mAb #14,962	ROS1	Thermofisher (Hs01053049_s1) (Chr.3: 181,711,924–181,714,436 on Build GRCh38)	[10, 40]
POU1F1, 3p11.2	Pit-1	Pit-1, D7; Santa Cruz		Thermofisher (Hs00230821_m1) (Chr.17: 34,255,277–34,257,203 on Build GRCh38)	[19, 24]
NR5A1, 9q33.3	SF-1	SF-1, EPR19744; Abcam		Thermofisher (Hs01124206_m1) (Chr.11: 64,764,604–64,779,043 on Build GRCh38)	[19, 24]
TBX19	T-Pit	TBX-19, CL6251; Sigma Aldrich		Thermofisher (Hs00193027_m1) (Chr.1: 168,281,040–168,314,426 on Build GRCh38)	[19, 24]
NES	Nestin	Abcam, monoclonal mouse, ICF-IHC	Pancreas	Thermofisher (Hs00707120_s1) (Chr.1: 156,668,763–156,677,397 on Build GRCh38)	[10, 13, 41]
PROM1	CD133	Abcam, polyclonal rabbit, ICF-IHC	Pancreas	Thermofisher (Hs01009259_m1) (Chr.4: 15,968,226–16,084,100 on Build GRCh38)	[10, 42]

Immunohistochemical evaluation of stem cell markers in tumours WDLD were compared with those meeting criteria for other WHO 2022 lineages. Subgroup analysis was performed to compare stem cell marker expression in WDLD tumours to those demonstrating clear lineage commitment: gonadotroph, lactotroph, somatotroph, mammosomatotroph, mixed somatotroph and lactotroph, thyrotroph, mature plurihormonal PIT-1 lineage and corticotroph, with the exclusion of tumours deemed “less mature” (Immature PIT-1 lineage and Acidophil stem cell tumours). There was one normal pituitary, obtained from a patient treated operatively for presumed pituitary tumour, with pathology yielding normal tissue only.

### RT-qPCR

Tumour samples were preserved immediately (fresh frozen) after surgery and stored within the Garvan Institute Pituitary Tumour Biobank. RNA was extracted from tumour samples. cDNA synthesis was performed using High-Capacity cDNA Reverse Transcription kit (Applied Biosystems). cDNA was diluted in RNAse free water (Thermofisher). RT-qPCR was performed of transcription factor and stem cell markers as per previously described methods utilising primers listed in Table 1 [24]. Purchased pooled normal pituitary cDNA (Takarabio: Tissue-specific human cDNA; Human Pituitary Gland QUICK-Clone cDNA from 12 male/female Caucasians aged 18–52) was used with each run of RT-qPCR. RT-qPCR results were reported as tumour mRNA expression relative to pooled normal pituitary mRNA expression. RT-qPCR evaluation of stem cell markers in WDLD tumours were compared with those meeting criteria for other WHO 2022 lineages.

### Statistical analysis

Results were presented as mean ± S.D. or median and interquartile range, as appropriate. Categorical variables were assessed by Chi-squared or Fisher’s exact test, while continuous variables which failed to satisfy parametric assumptions were assessed using the Mann-Whitney test. *P* < 0.05 was considered significant. Statistical analyses were carried out with SPSS version 27.

## Results

There were 88 samples included in the study in total, of which 53/88 underwent immunohistochemical analysis, 72/88 underwent RT-qPCR analysis and 38/88 underwent analysis using both methods. There was 1 normal pituitary that was part of the immunohistochemistry group, a 24-year-old female, operated as a presumed non-functioning pituitary tumour. The normal pituitary was included for descriptive immunohistochemistry results but not subsequent analysis. Baseline characteristics are summarised in Table 2. Clinical diagnoses were as follows: 41 non-functioning (NFPT) (normal pituitary included in this number, given the presumed diagnosis prior to surgery), 21 acromegaly (GHoma), 13 prolactinoma (PRLoma), 10 Cushing’s (ACTHoma) and 3 TSH-secreting tumours (TSHoma). Median age was 52 years (Interquartile range (IQR) 37.3–64.0).

**Table 2:** Baseline characteristics

	Complete cohort *n* = 88 (%)	IHC group *n* = 53 (%)	RT-qPCR group *n* = 72 (%)
Clinical diagnosis			
Normal pituitary NFPT Acromegaly Cushing’s disease Prolactinoma TSH-secreting	1 (1.1) 40 (45.5) 21 (23.9) 10 (11.4) 13 (14.8) 3 (3.4)	1 (1.9) 21 (39.6) 16 (30.2) 5 (9.4) 8 (15.1) 3 (5.7)	Pooled normal 34 (47.2) 19 (26.4) 6 (8.3) 10 (13.9) 3 (4.2)
Age (IQR)	52 (37.25-64.0)	50 (38.5–67.0)	52.5 (38.0–64.0)
Female sex	51 (58.0)	30 (56.6)	39 (54.2)
WHO 2022 diagnosis			
SF-1 lineage: gonadotroph PIT-1 lineage: lactotroph PIT-1 lineage: somatotroph PIT-1 lineage: mammosomatotroph PIT-1 lineage: mixed somatotroph and lactotroph PIT-1 lineage: thyrotroph PIT-1 lineage: mature plurihormonal PIT-1 lineage: immature PIT-1 lineage PIT-1 lineage: acidophil stem cell T-PIT lineage: corticotroph No distinct cell lineage: null cell No distinct cell lineage: plurihormonal Normal Not meeting criteria for WHO 2022	25 (28.4) 10 (11.4) 6 (6.8) 3 (3.4) 3 (3.4) 1 (1.1) 1 (1.1) 7 (8.0) 2 (2.3) 14 (15.9) 1 (1.1) 13 (14.8) 1 (1.1) 1 (1.1)	14 (26.4) 6 (11.3) 5 (9.4) 2 (3.8) 1 (1.9) 1 (1.9) 1 (1.9) 6 (11.3) 1 (1.9) 5 (9.4) 1 (1.9) 9 (17.0) 1 (1.9) 1 (1.9)	23 (31.9) 7 (9.7) 4 (5.6) 1 (1.4) 3 (4.2) 1 (1.4) 1 (1.4) 7 (9.7) 2 (2.8) 10 (13.9) 0 (0) 13 (18.1) Pooled normal 0 (0)
Invasiveness	30/86 (34.9)	18/53 (34.0)	25/70 (35.7)
Proliferative markers			
Ki67 ≥3% Mitotic count≥2 per 10 HPF^a^	21/86 (24.4) 19/86 (22.1)	11/52 (21.2) 11/52 (21.2)	15/70 (21.4) 14/70 (20)

^a^ HPF, high-powered field

### Immunohistochemistry group

There were 53 samples included in the analysis, including one normal pituitary. Baseline characteristics are summarised in Table 2. Clinical diagnoses were as follows: 21 NFPT, 16 GHoma, 8 PRLoma, 5 ACTHoma and 3 TSHoma. Complete immunohistochemistry was performed on normal pituitary. Stem cell markers, SOX2, CD133 and Nestin were negative in normal pituitary. It was then excluded from subsequent analysis. Median age was 50 (IQR 38.5–67.0) years. Cavernous and/or sphenoid sinus invasion was present in 18/53 (34%) patients. The WHO 2022 lineage type diagnoses were as follows: 14 gonadotroph (SF-1; all clinically NFPT); 6 lactotroph (Pit-1; all clinically PRLoma) ; 5 somatotroph (Pit-1; all clinically GHoma); 2 mammosomatotroph (Pit-1; 1 clinically GHoma and 1 clinically NFPT); 1 mixed somatotroph and lactotroph (Pit-1; clinically GHoma); 1 thyrotroph (Pit-1; clinically PRLoma); 1 mature PIT-1 lineage plurihormonal (Pit-1; clinically PRLoma); 6 immature PIT-1 lineage (Pit-1; 3 clinically GHoma and 3 clinically TSHoma); 1 acidophil stem cell (Pit-1; clinically NFPT); 5 corticotroph (T-Pit; all clinically ACTHoma); 1 null cell (clinically NFPT); 9 plurihormonal WDLD (7 clinically GHoma and 2 clinically NFPT). The 9 plurihormonal tumours WDLD were all strongly and diffusely positive for SF-1 and PIT-1. There was 1 tumour that did not meet criteria for WHO 2022 lineage type (clinically NFPT). This was a tumour that was negative for all transcription factors but expressed positive prolactin, TSH and growth hormone. There were 11 tumours with Ki67 ≥3% and 11 tumours with mitotic count ≥ 2/10 HPF; 15 tumours with Ki67 and/or mitotic count elevation and 7 tumours with both.

**Fig. 1:**
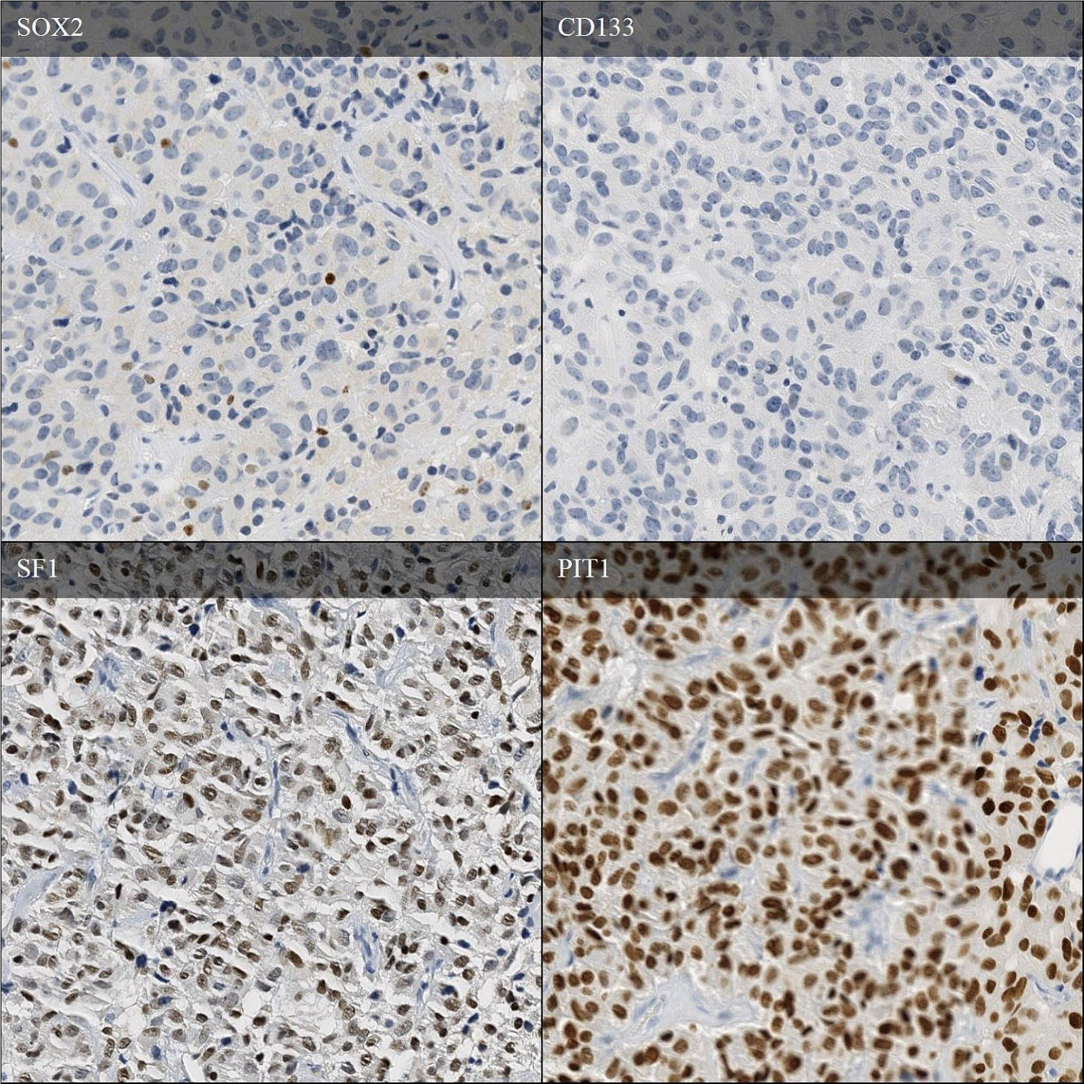
Plurihormonal WDLD tumour with strong diffuse SF1 and PIT1 expression throughout tumour, strong scattered SOX2 expression and negative CD133 expression (50X magnification)

Immunohistochemical evaluation of stem cell markers in tumours with no WDLD (*n* = 10) were compared to those meeting criteria for other WHO 2022 lineages (*n* = 42) (Table 3). SOX2 was positive in a higher proportion of WDLD tumours compared with those meeting WHO lineage criteria, 7/10 (70%) v 10/42 (23.4%), *P* = 0.005 (Fig. 1). Of SOX2 positive WDLD tumours the percentage of SOX2 positive cells ranged from 1 to 46% with 6/7 exhibiting strongly positive staining intensity (Table 4). By comparison, the SOX2 positive tumours with distinct cell lineage demonstrated a lower range percentage of SOX2 positive (1–4%), with variable intensity of expression. CD133 was positive in 2/10 WDLD tumours but 0/41 tumours meeting lineage criteria, *P* = 0.003. Of these two CD133 positive tumours, one co-expressed SOX2 and the other did not. Percent positivity ranged from 1 to 2% of tumour cells. There was no significant difference between Nestin immunopositivity across groups (2/9 v 18/40, *P* 0.209).

**Table 3:** Immunohistochemical evaluation of stem cell markers in tumours without distinct lineage differentiation compared with tumours meeting criteria for lineage-based classification (*n* = 52, IHC cohort with normal pituitary excluded)

	WDLD (*n* = 10)	Tumours meeting other WHO 2022 criteria (*n* = 42)	*P* value^b^
SOX2 positivity	7/10	10/42	0.005
CD133 positivity	2/10	0/41	0.003
Nestin positivity	2/9	18/40	0.209
Tumour invasiveness (sphenoid sinus &/or cavernous sinus)	4/10	14/42	0.690
Ki67 (≥3%)	0/10	11/42	0.064
Mitotic count (≥2/10 HPF)^a^	0/10	11/42	0.064
Ki67 and/or mitotic count elevation	0/10	15/42	0.025
Ki67 and mitotic count elevation	0/10	7/42	0.33

^a^ HPF, high-powered field

^b^*P* value evaluated using Chi-Square test and Fisher’s exact test, as appropriate based on the numbers included

**Table 4:** Tumours with SOX2 positivity on immunohistochemistry

Lineage	Clinical diagnosis	SOX2% positive cells	SOX2 intensity	SOX2 distribution
WDLD (SF-1, PIT-1)	GHoma	1	Strong	Scattered
WDLD (SF-1, PIT-1)	GHoma	3	Strong	Scattered
WDLD (SF-1, PIT-1)	GHoma	46	Strong	Mosaic
WDLD (SF-1, PIT-1)	GHoma	1	Moderate	Scattered
WDLD (SF-1, PIT-1)	GHoma	1	Strong	Scattered
WDLD (SF-1, PIT-1)	NFPT	8	Strong	Patchy
WDLD (null)	NFPT	35	Strong	Patchy
Gonadotroph (SF-1)	NFPT	1	Weak	Scattered
Gonadotroph (SF-1)	NFPT	1	Weak	Scattered
Lactotroph (PIT-1)	PRLoma	1	Weak	Scattered
Lactotroph (PIT-1)	NFPT	4	Strong	Scattered
Somatotroph (PIT-1)	GHoma	3	Strong	Patchy
Somatotroph (PIT-1)	GHoma	1	Strong	Scattered
Immature PIT-1 lineage (PIT-1)	GHoma	1	Strong	Scattered
Immature PIT-1 lineage (PIT-1)	GHoma	4	Strong	Scattered
Immature PIT-1 lineage (PIT-1)	TSHoma	1	Strong	Scattered
Corticotroph (T-PIT)	ACTHoma	1	Strong	Scattered

Subgroup analysis was performed to compare stem cell marker expression in WDLD tumours to those demonstrating clear lineage commitment (*n* = 35) (Table 3). For this analysis, we excluded all Immature PIT-1 lineage (*n* = 6) and Acidophil stem cell tumours (*n* = 1). The aforementioned findings were upheld on this analysis, with SOX2 and CD133 both more expressed in WDLD tumours than those with committed lineage (p 0.006 and 0.056). We also compared both Immature PIT-1 lineage and Acidophil stem cell tumours with those with clear lineage commitment and found no difference in immunoexpression of stem cell markers (p 0.143 and 1.00 for SOX2).

Comparing WDLD with other tumours meeting lineage criteria we found no significant difference in tumour invasion between groups. There were no WDLD tumours with elevation in proliferative markers (Ki67 or mitotic count). In fact, 15/42 (36%) of tumours meeting lineage criteria had elevation in Ki67 or mitotic count (*p* = 0.025). Tumour invasiveness and proliferation were not associated with stem cell marker expression. Elevation of mitotic count was significantly associated with immunoexpression of Nestin, but not SOX2 or CD133.

### RT-qPCR group

There were 72 samples that underwent RT-qPCR analysis. Baseline characteristics of the RT-qPCR group are summarised in Table 2. Clinical diagnoses were as follows: 34 NFPT, 19 GHoma, 6 ACTHoma, 10 PRLoma and 3 TSHoma. Median age was 52.5 (IQR 38.0–64.0). The WHO 2022 lineage type diagnoses were as follows: 23 gonadotroph, 7 lactotroph, 4 somatotroph, 1 mammosomatotroph, 3 mixed somatotroph and lactotroph, 1 thyrotroph, 1 mature plurihormonal, 7 immature PIT-1 lineage, 2 acidophil stem cell, 10 corticotroph, 13 plurihormonal tumours WDLD. There were 15 tumours (21.4%) with elevated Ki67 and 14 tumours (20%) with elevated mitotic count; overall 20 (27.8%) had elevation of Ki67 and/or mitotic count.

RT-qPCR evaluation of stem cell markers in WDLD tumours (*n* = 13) were compared with those meeting criteria for other WHO 2022 lineages (*n* = 59). There was no significant difference in relative mRNA expression of SOX2 (91.7 (24.9-172.9) v 59.2 (21.0-111.3), *p* = 0.244), Nestin (17.7 (6.3–34.6) v 14.8 (6.7–28.5), *p* = 0.884) or CD133 (4.2 (0.8–8.4) v 4.5 (1.3–26.1), *p* = 0.487) in the WDLD tumours compared with tumours meeting lineage criteria. Within the WDLD tumours, only 1/13 tumours had elevation of Ki67 and 0/13 tumours had elevation of mitotic count; the between group difference in proliferative marker expression did not reach statistical significance.

### Comparison of transcription factor expression by IHC and RT-qPCR

Within the subgroup of tumours with complete IHC and RT-qPCR (*n* = 38), transcription factor protein expression on IHC was associated with relative mRNA expression on RT-qPCR (Table 5). In SF-1 lineage tumours (*n* = 12), relative SF-1 mRNA expression was elevated, but not significantly, whereas both PIT-1 and T-PIT expression were significantly suppressed. In PIT-1 lineage tumours (*n* = 16), relative PIT-1 mRNA expression was significantly elevated and T-PIT significantly suppressed. SF-1 expression was also present in Pit-1 lineage tumours but not statistically significantly higher than normal pituitary. In the single T-PIT lineage tumour, relative T-PIT mRNA expression was markedly elevated, PIT-1 was suppressed, and some SF-1 expression present. In the plurihormonal WDLD tumours (*n* = 9), there was no significant difference in relative mRNA expression of the transcription factors compared to normal pituitary.

**Table 5:** Association of relative mRNA expression via RT-qPCR^a^ versus tumour lineage as determined by immunohistochemistry (*n* = 38)

	Lineage, determined by immunohistochemistry							
	SF-1 lineage (*n* = 12)	*P* value^b^	PIT-1 lineage (*n* = 16)	*P* value^b^	T-PIT lineage (*n* = 1)	*P* value^b^	Plurihormonal, WDLD (*n* = 9)	*P* value^b^
SF-1	6.0 (3.7–9.6)	0.679	5.4 (4.5–12.1)	0.812	7.2	0.545	14.9 (6.5–43.5)	0.063
PIT-1	0.0 (0.0–0.0)	< 0.001	23.7 (12.4–47.4)	< 0.001	0.0	0.364	13.0 (3.3–89.6)	0.208
T-PIT	0.5 (0.4–0.6)	< 0.001	1.8 (1.0-2.9)	0.005	292.3	0.091	0.7 (0.5–2.7)	0.919
SOX2	35.0 (5.7–78.8)	0.227	45.7 (18.2–105.0)	0.329	66.0	0.545	130.3 (20.0-185.0)	0.142
Nestin	11.1 (3.4–14.2)	0.347	9.7 (4.8–28.5)	0.559	18.3	0.273	10.7 (5.1–8.4)	0.686
CD133	4.1 (0.4–38.8)	0.586	4.0 (1.4–18.5)	0.589	4.5	1.000	4.2 (0.8–8.4)	0.840

^a^ Relative mRNA expression by RT-qPCR, compared with normal pituitary. Values expressed as median, interquartile range

^b^*P* value determined by Mann-Whitney Test

## Discussion

This is the first study to investigate and characterise tumours with “without distinct lineage differentiation”, as described in the 2022 WHO classification, by examination of stem cell marker expression and association with clinical parameters. Compared to pituitary tumours meeting clear lineage criteria, we found WDLD tumours more frequently expressed stem cell markers SOX2 and CD133 on IHC, the majority of which exhibited strong SOX2 intensity. Immature PIT-1 lineage tumours and Acidophil stem cell tumours did not demonstrate increased immunoexpression of stem cell markers. On RT-qPCR analysis, there was slightly higher relative mRNA expression of SOX2 and Nestin in tumours WDLD compared with others which did not reach statistical significance. Within both IHC and RT-qPCR groups, proliferative markers tended to be more frequently elevated in tumours meeting WHO 2022 lineage criteria, collectively but not individually reaching statistical significance. Larger numbers of tumours are required to determine if the non-significant results represent a type 2 statistical error.

Comparison between transcription factor expression by IHC and RT-qPCR demonstrated mixed results with regards to mRNA relative expression, however grossly reflected the immunohistochemistry findings without reaching statistical significance. Our results are consistent with previous findings that gonadotroph tumours expressed similar levels of SF-1 mRNA compared with other tumour types [24]. We hypothesise that this may reflect inclusion of normal cells within tissue collection for DNA extraction, skewing results. Additionally, there is evidence to suggest that discrepancy between gene and protein expression may relate to other factors such as miRNAs and mRNA stability. Based on our results and previous studies, we consider IHC to be the more reliable method for interpretation of transcription factors and stem cell markers [24].

In 2022, the WHO Classification defined a new type of tumour, “plurihormonal tumours WDLD”. By definition, these express multiple combinations of lineage commitment transcription factors, in a monomorphous population of cells [22]. Despite the inclusion in the WHO Classification, these tumours remain to be fully characterised and have henceforth been described only in case reports and one cohort study [20, 21, 23, 25]. The first reported a tumour co-expressing SF-1 and PIT-1 and proposed that multiple cellular commitment transcription factors within one population may arise in context of a collision tumour [21]. Subsequently, the same group reported another tumour co-expressing SF-1 and PIT-1, this time proposing that co-expression of multiple transcription factors may arise in a “poorly differentiated stem cell tumour” [20]. To our knowledge, this is the first study to characterise such tumours, and to evaluate stem cell markers on whole tumour sections.

Pituitary tumours are increasingly recognised as heterogenous neoplasms, with varying degrees of cellular maturity and plasticity. There is a growing body of literature proposing immaturity of “immature PIT-1 lineage” tumours, previously known as “silent subtype 3” and “PIT-1 plurihormonal” tumours [26–30]. Studies largely predate the adoption of transcription factor-based classification, necessitating further expansion of the literature, with more contemporary diagnostic techniques. Nonetheless, evidence suggests that these tumours are associated with progressive disease. Moreover, authors have postulated that these tumours might demonstrate a degree of “immaturity”, based on histopathological characteristics [26–28, 31–33]. However, in this study, we did not find higher expression of stem cell markers in these compared with other tumours, indicating that the term “immature” may not be an accurate reflection of tumour biology. To date, no other studies have investigated whether these tumours express stem cell markers.

There is a growing body of literature investigating cellular plasticity in pituitary tumours. In a recent study, SF-1 immunoexpression in gonadotroph tumour follicular cells was proposed to reflect neuroplasticity, specifically transformation of neoplastic cells into follicular cells [34]. Cancer stem cell theory postulates that tumours arise from cancer stem cells (CSC), which maintain by self-renewal and have multilineage potential, accounting for intratumour heterogeneity, persistence and recurrence [35]. CSC theory has been illustrated in other malignancies and more recently, there have been a number of human pituitary studies that have isolated cells fulfilling some or all of the CSC criteria [10–15]. However, recent mouse model studies suggest that stem cells do not directly generate tumourigenesis in the pituitary, rather they become activated in tumourigenesis, then fuel development and growth [35–37]. One study reported a stem cell compartment of SOX2 expressing cells, which was expanded in the early phase of the tumourigenic process [38]. Another study utilised immunofluorescence in human pituitary tumours to demonstrate expression of stem cell markers, SOX2 and SOX9, in a variety of clinical tumour types. SOX2 immunofluorescence was observed in tumours of different hormonal types (21/21) to varying degrees (0.05–38.24%), noting that transcription factors were not assessed. Stem cell markers, SOX2 and SOX9, were associated with increased proliferative status [3]. Interestingly, SOX2 immunofluorescence was detected in all (21/21) tumours, whereas SOX2 immunopositivity was detected in 17/55 tumours in the present study [3]. Differential expression between studies may be explained at least in part by the higher sensitivity of immunofluorescence compared with immunohistochemistry analysis. Another recent study compared gonadotroph tumours with varying levels of SF-1 IHC expression. SOX2 was upregulated on RNA sequencing in those tumours with low or patchy SF-1 expression when compared with those with diffuse SF-1 expression. Authors postulated that low SF-1 expression in gonadotroph tumours may represent intratumoural heterogeneity and lesser differentiation [39]. Further studies will help to refine IHC methods used to assess stem cells markers in human pituitary tumour tissue. Pituitary tumour stem cell marker expression has been associated with increased markers of proliferation, chemokines and cytokines, including IL-6, but not with clinically aggressive or metastatic disease, supporting a role in early tumourigenesis [3]. Our study identified increased stem cell marker but not proliferative marker expression in WDLD tumours, thereby supporting a possible role of stem cells in tumour formation and pituitary tumour cell differentiation. However, further studies are required to elucidate the role of stem cell marker positive cells within these tumours.

There are several strengths of this study. It is the first to characterise a series of WDLD Tumours and to investigate a possible role for stem cells in such tumours. We evaluated transcription factors and stem cell markers on whole sections, using digital imaging analysis technology and three pituitary pathologists with expertise in this field. We compared immunohistochemical findings with mRNA expression via RT-qPCR, though in our view, the latter appears to be a less robust method of assessment, both due to inclusion of bulk cells and variability between protein and mRNA expression. Future studies with spatially resolved profiling will help to elucidate stem cell marker expression and distribution. There are limitations of this study that must be acknowledged. Although we did not isolate tumour cells to confirm stem cell characteristics, previous human pituitary studies have demonstrated such findings in association with immunopositivity for markers CD133, SOX2 and Nestin. The selection of tumour samples was based on quality of cases, DNA and whole sections for the purposes of our analysis. This is associated with inherent selection bias and precludes prolonged follow-up. Future studies are required to investigate clinical outcomes in these tumours.

In conclusion, our study has characterised a novel type of pituitary tumour without distinct lineage differentiation. We have shown that WDLD tumours exhibit a higher expression of the stem cell marker SOX2, suggesting a possible involvement of stem cells in their development. Further studies are required to expand our characterisation of such tumours and to better understand the role of stem cells in early tumourigenesis of WDLD tumours. By expanding our knowledge in this field, we aim to develop scope for targeted therapies that may be used to treat tumours in early stages.
